# Resistance to penicillin of Staphylococcus *aureus *isolates from cows with high somatic cell counts in organic and conventional dairy herds in Denmark

**DOI:** 10.1186/1751-0147-48-24

**Published:** 2006-11-24

**Authors:** Torben W Bennedsgaard, Stig M Thamsborg, Frank M Aarestrup, Carsten Enevoldsen, Mette Vaarst, Anna B Christoffersen

**Affiliations:** 1 Department of Animal Health, Welfare and Nutrition, Danish Institute for Agricultural Sciences, 8830 Tjele, Denmark; 2 Department of Veterinary Pathobiology, The Royal Veterinary and Agricultural University, 1870 Frederiksberg C, Denmark; 3 Danish Institute for Food and Veterinary Research, 1790 Copenhagen V, Denmark; 4 Department of Large Animal Sciences, The Royal Veterinary and Agricultural University, 1870 Frederiksberg C, Denmark

## Abstract

**Background:**

Quarter milk samples from cows with high risk of intramammary infection were examined to determine the prevalence of *Staphylococcus aureus *(SA) and penicillin resistant SA (SAr) in conventional and organic dairy herds and herds converting to organic farming in a combined longitudinal and cross-sectional study.

**Methods:**

20 conventional herds, 18 organic herds that converted before 1995, and 19 herds converting to organic farming in 1999 or 2000 were included in the study. Herds converting to organic farming were sampled three times one year apart; the other herds were sampled once. Risk of infection was estimated based on somatic cell count, milk production, breed, age and lactation stage.

**Results:**

The high-risk cows represented about 49 % of the cows in the herds. The overall prevalence of SA and SAr among these cows was 29% (95% confidence interval: 24%–34%) and 4% (95% confidence interval: 2%–5%) respectively. The prevalence of penicillin resistance among SA infected cows was 12% (95% confidence interval: 6%–19%) when calculated from the first herd visits. No statistically significant differences were observed in the prevalence of SAr or the proportion of isolates resistant to penicillin between herd groups.

**Conclusion:**

The proportion of isolates resistant to penicillin was low compared to studies in other countries except Norway and Sweden. Based on the low prevalence of penicillin resistance of SA, penicillin should still be the first choice of antimicrobial agent for treatment of bovine intramammary infection in Denmark.

## Background

Staphylococcus *aureus *(SA) is the most commonly occurring pathogen in udder quarters with elevated somatic cell counts (SCC) in Denmark and accounts for approximately 50% of the intramammary infections of lactating cows [1]. Experimental infections with SA have shown that infected cows develop high SCC, though both the SCC and the number of bacteria shed in the milk vary considerably both between cows and within quarters over time [2]. The control of SA infections in dairy herds often includes a combination of preventive measures to reduce the number of new infections, dry cow treatment of all cows with antibiotics, treatment of infected animals, and culling of chronically infected animals [3,4]. Frequent use of antibiotic treatment in dairy cows has been proposed to comprise a risk for development of or selection for SA resistant to antibiotics [5]. However, results of susceptibility patterns for commonly used antibiotics indicate that the prevalence of β-lactamase producing SA which are resistant to penicillin seems to have remained at a fairly constant level (40–60%) for the last twenty years. Norway, Sweden and Denmark are exceptions because they have had a consistently lower proportion of penicillin resistant isolates (10–20%) than other countries [6,7]. Comparison of susceptibility data from different surveys is complicated because both the selection of isolates and the methods used for susceptibility testing differ. Often a few clones of SA dominate in the single herd due to the contagious nature of the bacteria. Therefore, surveys only including few herds might provide invalid estimates of the general prevalence [1,8,9]. Similarity of phage types among quarters from the same cow and analysis of infection patterns in the quarters of a cow indicate that the multiple SA isolates from the individual cow are most often a result of an infection from the initially infected gland, and consequently isolates from the same cow cannot be regarded as independent [1,10].

The cure rate after therapy for both clinical and subclinical mastitis has been shown to be lower for β-lactamase-positive *S. aureus *compared to β-lactamase-negative *S. aureus *strains [11-14].

Antibiotic resistance is a major concern for consumers due to the zoonotic potential. In Denmark, prophylactic use of antibiotics is prohibited, and dry cow treatment can only be performed legally in cows with an actual or recent case of clinical mastitis or a positive bacteriological culture.

The organizations for organic agriculture have imposed additional restrictions on the use of antibiotics as an incentive to mitigate the risk of antibiotic resistance and to motivate the farmers to achieve a good herd health without the use of antibiotics. However, it has not been shown whether these initiatives have affected the occurrence of antibiotic resistance in the organic herds.

The aim of this study was to compare the prevalence of SA and penicillin resistant SA (SAr) in conventional and organic dairy herds in Denmark and to monitor the prevalence of SA and SAr in the first two years after conversion from conventional to organic milk production.

## Methods

### Collection of samples

Twenty conventional herds, 18 herds which converted to organic production at least five years before the start of the study (old organic) and 19 herds converting in 1999 or 2000 were included in the study as part of a larger project concerning udder health (The Kongeaa project) [15]. All herds were located in the southwestern part of Denmark. The conventional and old organic herds were sampled once between March and June 2000. In all herds, quarter milk samples were collected from 30 cows with high somatic cell counts. The criteria for sampling were an estimated risk of infection based on the history of SCC, breed, and calving number of the individual cow [16]. If more than 30 cows had an estimated risk of infection above 50 %, 30 cows were sampled at random among these cows, based on a computer-generated list. In herds with less than 30 cows with a score for infection risks above 50 %, the 30 cows with the highest estimated infection risk were sampled. Samples were collected aseptically by technicians employed by the Danish Dairy Board according to standard procedures [17].

Data on milk production and SCC from monthly test days and information on breed, age, and calving number were available from the Danish Cattle Database for at least one year prior to sampling for all sampled herd and for an additional group of 109 herds enrolled in the entire Kongeaa project. Recording of veterinary treatments in the central database was crosschecked with registrations in the herds for the 57 herds in this study.

To evaluate the consequences of only sampling herds with a high infection risk a dataset consisting of herd tests with quarter milk samples from all cows in 125 herds Danish dairy herds collected between 1995 and 2000 was used

### Laboratory procedures

Laboratory examinations were performed according to standard procedures by the Danish Cattle Health Laboratory, Ladelund [17]. 10 μl of milk was streaked on to blood agar plates supplemented with aesculin and incubated at 37°C for 18–24 hours. SA was identified based on morphology and β-toxin production. Penicillin resistance of SA was tested on blood agar plates with 1 IU penicillin per ml. The results of the laboratory examinations were given as a microbial diagnosis and California Mastitis Test (CMT) scores of the quarter milk sample. CMT was measured on a five-point scale with 1 as completely negative, 3 as clearly positive and 5 as maximum. For CMT ≤ 2 at least 5 colony forming units (CFU) SA per plate were regarded as positive, whereas for CMT > 2 or milk with visible changes, growth of more than 2 CFU were regarded as positive.

### Statistical analysis

Descriptive statistics for herd size, milk production, estimated bulk tank somatic cell counts, based on the individual cow yield, SCC and mastitis treatments for the last year prior to sampling were calculated (Table 1) Statistical significance of differences in mastitis treatments between the herds in the study and the reference group of 109 herds was not made since the data from the 109 herds were not validated.

**Table 1:** Herd size, production and herd health in Danish dairy herds. Characteristics of herd groups in the study.

	No. herds	End of one year study period	Herd size Cow years/year	Calculated bulk tank somatic cell count	Milk production Kg ECM/day	Mastitis treatments % cows treated/cow year	% of cows with infection risk > 50%
Conventional	20	03–2000	83	283	25.7^a^	74^a^	42
Organic before 1995	18	03–2000	88	296	22.1^c^	48^b^	38
Converting herds before conversion	19	04–1999 or 04–2000	87	317	24.6^a,b^	61^a,b^	42
Converting herds first year after conversion	19	04–2000 or 04–2001	101	337	23.3^c^	52^a,b^	44
Converting herds second year after conversion	19	04–2001 or 04–2002	107	327	23.8*	48^a,b^	45
Conventional herds in full research project	109	03–2000	85	309	24.4^b^	55*	43

Different letters: P < 0.05

*No comparison made

Only results from cows with an infection risk above 50 % was included in the analysis. Data was analyzed by logistic regression. The analysis was performed with SAS 8.2 software (SAS institute, Cary, USA) using the procedure PROC MIXED with the GLIMMIX macro with the REML algorithm and restricted quasi-likelihood method. Isolation of penicillin resistant SA in at least one quarter milk sample from a cow was used as outcome as a binary variable with the logit link function. Herd was introduced as a random variable in a hierarchical model. A categorical variable for the five herd groups: conventional, old organic, converting herds year 0, converting herds year 1 and converting herds year 2 was introduced to test differences between herd groups. The same analysis was performed with the isolation of any SA in at least one quarter milk sample from a cow as outcome. Based on the models, the differences in the prevalences of penicillin resistant SA and total SA were tested (Table 2). Due to underdispersion in the model for SAr, differences in the isolation of at least one SAr at herd level were also compared using χ^2^-tests.

**Table 2:** Herd averages of prevalence of S. *aureus *and penicillin-resistant S. *aureus *in different Danish herd groups of cows with high risk of infection. (no. cows = 2311)

	No. herds	No. of cows tested	% of cows with SA	No. herds with SAr	% of herds with SAr	% of cows with SA	% of cows with SA with SAr isolates
Conventional (2000)	20	493	23^a^	8	40	2	8
Organic before 1995 (2000)	18	391	25^a,b^	7	38	6	22
Converting herds before conversion (1999 or 2000)	19	498	39^b^	7	37	3	10
Converting herds one year after conversion	19	481	36^a,b^	4	21	1	8
Converting herds two years after conversion	19	493	36^a,b^	8	36	3	7

Different letters: P < 0.05

## Results

### Herd characteristics

The differences in somatic cell counts and number of mastitis treatments were not statistically significant (Table 1). Milk production and the prevalence of mastitis treatment in the conventional group were significantly higher than in the old organic and the converting herds after one year of organic production; it was also significantly higher than in the larger group of 109 herds enrolled in the entire project. The average herd size of the herds converting to organic farming was larger although the difference was not statistically significant because only a smaller group of herds was enlarged.

### Prevalence of SA and SAr

SA was isolated from one or more quarter milk samples from 749 out of 2,311 cows (32%). Out of these SAr were isolated from 74 cows (10%). SA was isolated from at least one cow at all herd visits except in five herds (two conventional, one old organic, one converting herd year 0 and one converting herd year 1). At six herd visits < 10 cows had an infection risk > 50%. At 18 herd visits < 20 cows had an infection risk > 50 percent.

In the herds converting to organic farming the prevalence of SA infection at cow level was significantly higher before conversion (39%) compared to the conventional group (23%) (P = 0.03). The differences between all other herd groups were non-significant. SAr were only found at 36% of the herd visits. No significant differences where found in the prevalence of SAr between the herd groups. The model for SA infections fitted the binomial distribution closely. However, the distribution of the SAr data set showed severe under-dispersion (φ = 0.5) probably due to the large number of herds without any isolates and the inter/dependence between isolates within the herds resulting in a few herds with very high prevalence of resistance. Tests of differences between herd groups on isolation of at least one SAr on herd level using did not show any significant differences.

Due to the underdispersion the model estimated the prevalence of SAr resistance lower than the simple average. The average proportion of SA infected cows that had at least one isolate resistant to penicillin was estimated to 6 % (95% confidence interval: 3%–12%) when calculated from the first herd visits compared to the simple herd average of 12% (95% confidence interval: 6%–19%).

### Changes over time

The 19 herds converting to organic farming were tested three times one year apart. SAr was found at least once in 11 of the herds (61%) and only in two herds resistant isolates were found at all three visits. In five of the herds SAr was only found at one visit. In one herd the prevalence of SAr infected cows changed from none to 32% of the tested cows within one year. In another herd the prevalence of SAr changed from 23% to 0% over two years while the overall prevalence of SA only decreased from 80% to 60%.

## Discussion

### Herd characteristics

The evaluated parameters for production, udder health and disease treatments are comparable to a larger study of production, udder health and disease treatments in organic and conventional herds in Denmark. In that study only organic herds converted before 1990 showed lower calculated bulk tank SCC and fewer mastitis treatments [18].

### Prevalence of SA and SAr

It is not possible to estimate the prevalence at herd level with the chosen sample scheme because the prevalence of infection among cows with low infection risk is not known. However, an analysis of a data set of 125 herd tests where quarter milk samples were taken from all cows showed that about 80% of both the SA and SAr isolates from all cows were found in the forty-eight percent of the cows that had an infection risk above 50% (unpublished). Based on that finding it appears that most SA infected cows were identified by the chosen sample scheme and that the sample scheme allowed identification of penicillin resistant and susceptible SA equally well.

### Changes over time

The changes from year to year in the herds converting to organic farming indicate that even though the SA infections might be dominated by a single dominant clone(s) at a given time, new clones may take over the dominant position in relative short time. The low prevalence of SAr combined with the relative low sensitivity of milk samples to detect SA infections estimated to about 75% [2,19] might also explain some of the variation in prevalence. In some of the herds a large number of animals were bought from other herds during the study period. These animals might also have influenced the prevalence and the strains of SA found in the herds.

The small non-significant differences in SCC and the use of mastitis treatments and the significant differences in milk production between organic and conventional herds did not result in any difference in the neither the prevalence of SA infections nor the proportion of SA being resistant to penicillin. The prevalence of SAr in the group of old organic herds was strongly influenced by a few herds with very high proportion of resistant SA.

### Comparison with other studies

The level of resistance in SA from intramammary infections has usually been reported as a proportion of the total number of SA. The large proportion of herds with no penicillin resistant SA isolates indicates that the occurrence of SAr must be seen as a herd problem at the present low overall prevalence of resistant isolates. The resistant isolates found in single herds probably represent the same clone. Despite large uncertainty on the estimates, the results are in agreement with previous Danish studies. Penicillin resistance was found in 14% to 22% of the SA isolates from milk samples examined at the Danish Veterinary Institute (DVI) from 1994 to 2001 [6,20]. From 1963 to 1983 isolates from different surveys and routine diagnostic samples showed a prevalence of penicillin resistance between 3.1% and 7.2%, from 1983 to 1988 the prevalence varied from 7.0 to 11.4%. The prevalence of penicillin resistance found at DVI from 1994 to 2001 was higher than the results of this study. This result is biased, because the material at DVI is mainly based on samples from cows with clinical mastitis.

Most recent publications indicate a decrease in penicillin resistance of SA in several countries during the last 10 years. In Belgium, the prevalence of penicillin resistance of SA from clinical and subclinical mastitis was 38% in 1971, 81% in 1977 and decreased to 51% in 1996 [21]. In France, 64% and 49% of the isolates showed penicillin resistance in 1990–1993 and 1994–2000 respectively [22,23]. In Germany 62% of the isolates from the western part and 30% of the isolates from the eastern part of the country were resistant to penicillin in 1991–1992 and 52% of the isolates from the whole country in 1997 [24]. In Michigan, USA the prevalence of resistant isolates was 62% in 1994 and 42% in 1999 and a decreasing linear trend in data from 1994 to 1999 was statistically significant [25]. In 2001, 18% of the isolates from 99 Swedish cows with subclinical or chronic mastitis were resistant to penicillin [26]. In 2001 in Norway, 11% of 3,557 SA isolates from quarter milk samples from herd tests and 5% of the isolates from moderate or severe clinical mastitis were resistant to penicillin. In all years since 1980, < 18% of the SA isolates from herd tests have been resistant to penicillin [27]. Compared to these studies, the prevalence of penicillin resistance as demonstrated in our study is low in Denmark. It has been suggested that penicillin should be the first choice of antimicrobial agent for treatment of udder infections supposed to be caused by gram-positive bacteria when the prevalence of penicillin resistant SA is below 10% in a herd [6]. In 43 of the 57 herds in this study, the prevalence of resistant isolates from cows infected with SA was below this level.

## Conclusion

No difference in prevalence of penicillin resistant SA or in the proportion of SA resistant to penicillin was found between conventional and old organic herds or before and after converting to organic farming. The overall prevalence of SAr was low, at about 4% of the cows with high infection risk and the proportion of resistant isolates at about 12%. The low level of resistance makes penicillin a good choice for treatment of intramammary infections in Danish dairy herds. However, based on the changes in prevalence over time and the possible differences in strains causing high SCC and clinical mastitis milk, it can be recommended to monitor the antimicrobial susceptibility on a regular basis. A regular sampling at the herd level will also provide the necessary information for choosing the most effective preventive measures for controlling udder infections in general.

## Abbreviations

CMT: California Mastitis Test

SA: *Staphylococcus aureus*.

SAr: Penicillin-resistant SA.

SCC: Somatic cell count.

## Competing interests

The author(s) declare that they have no competing interests.

## Authors' contributions

TWB, FMA, SMT and CE have been involved in the initial design of the study and protocols. ABC has been responsible for the microbiological work in the laboratory at the Cattle Health Laboratory. TWB has been the main responsible for data analysis in coorporation with TWB, CE, SMT and FMA. All authors have contributed substantially to the editing of the manuscript.
